# Integrating biochemical and anatomical characterizations with transcriptome analysis to dissect superior stem strength of ZS11 (*Brassica napus*)

**DOI:** 10.3389/fpls.2023.1144892

**Published:** 2023-05-09

**Authors:** Zhengshu Tian, Xinfa Wang, Xiaoling Dun, Ze Tian, Xiaoxue Zhang, Jinfeng Li, Lijun Ren, Jinxing Tu, Hanzhong Wang

**Affiliations:** ^1^ Key Laboratory of Biology and Genetic Improvement of Oil Crops, Oil Crops Research Institute of the Chinese Academy of Agricultural Sciences, Ministry of Agriculture, Wuhan, China; ^2^ College of Plant Science and Technology, Huazhong Agricultural University, Wuhan, China; ^3^ Industrial Crops Institute, Yunnan Academy of Agricultural Sciences, Kunming, China; ^4^ Hubei Hongshan Laboratory, Wuhan, China

**Keywords:** *Brassica napus*, stem strength, rind penetrometer resistance, cell wall components, S-adenosylmethionine

## Abstract

Stem lodging resistance is a serious problem impairing crop yield and quality. ZS11 is an adaptable and stable yielding rapeseed variety with excellent resistance to lodging. However, the mechanism regulating lodging resistance in ZS11 remains unclear. Here, we observed that high stem mechanical strength is the main factor determining the superior lodging resistance of ZS11 through a comparative biology study. Compared with 4D122, ZS11 has higher rind penetrometer resistance (RPR) and stem breaking strength (SBS) at flowering and silique stages. Anatomical analysis shows that ZS11 exhibits thicker xylem layers and denser interfascicular fibrocytes. Analysis of cell wall components suggests that ZS11 possessed more lignin and cellulose during stem secondary development. By comparative transcriptome analysis, we reveal a relatively higher expression of genes required for S-adenosylmethionine (SAM) synthesis, and several key genes (*4-COUMATATE-CoA LIGASE*, *CINNAMOYL-CoA REDUCTASE*, *CAFFEATE O-METHYLTRANSFERASE*, *PEROXIDASE*) involved in lignin synthesis pathway in ZS11, which support an enhanced lignin biosynthesis ability in the ZS11 stem. Moreover, the difference in cellulose may relate to the significant enrichment of DEGs associated with microtubule-related process and cytoskeleton organization at the flowering stage. Protein interaction network analysis indicate that the preferential expression of several genes, such as *LONESOME HIGHWAY* (*LHW*), *DNA BINDING WITH ONE FINGERS* (*DOFs*), *WUSCHEL HOMEOBOX RELATED 4* (*WOX4*), are related to vascular development and contribute to denser and thicker lignified cell layers in ZS11. Taken together, our results provide insights into the physiological and molecular regulatory basis for the formation of stem lodging resistance in ZS11, which will greatly promote the application of this superior trait in rapeseed breeding.

## Introduction

Rapeseed (*Brassica napus*) is one of the most important oilseed crops that is utilized as edible oil for humans, as feeding protein for animals, and as sustainable biomass energy for industry (Wei et al., 2017; Shao et al., 2022). In view of the importance of this oil crop, continuously improving the crop yield and quality of oil remains the most important breeding objectives of rapeseed. Although the per unit yield of rapeseed has been greatly improved, lodging-induced damages in the production and quality of rapeseed results in huge economic losses every year. For instance, lodging can reduce the yield of rapeseed about 20 - 46% at the flowering stage (Kendall et al., 2017). Stem lodging, which is a major type of crop lodging, not only alters the canopy structure but also affects the photosynthetic efficiency of leaves (Kuai et al., 2016; Weng et al., 2017). Additionally, stem breaking may impede the transfer of nutrients and the accumulation of oil components in rapeseed (Armstrong and Nicol, 1991; Berry and Spink, 2009). Consequently, improving the lodging resistance of the stem has become an important task for rapeseed breeding.

The mechanical strength of the stem is intimately related to lodging resistance since this serves as the main mechanical support tissue for the aboveground plant. Numerous studies have shown that rind penetrometer resistance (RPR) and stem breaking strength (SBS) are reliable indicators for evaluating stalk lodging resistance (Hu et al., 2012; Hu et al., 2013; Liu et al., 2020; Zhang et al., 2020; Hou et al., 2022). Genetic analysis of the RPR trait in maize showed that it is affected by several minor QTL loci, some of which perform regulatory roles during multiple growth periods (Liu et al., 2020). SBS can be described as the maximum load exerted to the breaking (F max), the breaking moment (M max) and the critical stress (σ max) parameters. Genetic force analysis indicates that F max contributed the most to the mechanical strength of the stem (Hu et al., 2012; Hu et al., 2013). Several studies have revealed that some genetic loci in SBS and RPR shared the same flanking markers or overlapping confidence intervals with lignin or cellulose concentration-related loci, suggesting that these traits may be influenced by some common genetic factors (Hu et al., 2012; Hou et al., 2022; Shao et al., 2022).

The chemical composition of the cell wall has been shown to be highly positively correlated with stem mechanical strength (Zhang et al., 2020; Bisht et al., 2022; Hu et al., 2022). Lignin is one of the most important cell wall components that affect stem mechanical strength. In plants, the biosynthesis of lignin is mainly started from L-Phenylalanine. The biosynthesis pathway is regulated by a series of enzymes with oxidized and reduced activity (such as *4CL*, *F5H* (*FERULATE 5-HYDROXYLASE*), and *CCR*) and methylation activity (such as *COMT*, and *CCoAOMT* (*CAFFEOYL-COENZYME A O-METHYLTRANSFERASE*)) (Dixon and Barros, 2019; Li et al., 2022). In *Arabidopsis thaliana*, simultaneous mutations of *4CL* and *COMT* resulted in a decreased lignin content (Vries et al., 2018). And downregulating the expression of *CCR* and *CAD* (*CINNAMYL ALCOHOL DEHYDROGENASE*) greatly inhibited the biosynthesis of lignin in transgenic tobacco plants (Chabannes et al., 2001). Moreover, overexpression of *TaCOMT-3D* in wheat can enhance the accumulation of lignin lines and improved stem mechanical strength successfully (Wang et al., 2018). Consequently, optimization of cell wall content by regulating the expression of genes related to lignin synthesis suggests a great potential application in stem strength improvement.

Cellulose is another important factor contributing to stem strength. In rice, several *brittle culm* (*bc*) mutants showed decreased cellulose concentration in the secondary cell wall, with a corresponding decrease in mechanical strength of the culm (Li et al., 2003; Yan et al., 2007; Kotake et al., 2011; Sato-Izawa et al., 2020). In maize, downregulation of the *stiff1* resulted in the upregulation cellulose and lignin contents, leading to the greater stalk strength (Zhang et al., 2020). More importantly, the locus of *stiff1* has been applied in breeding for stalk improvement because the promoter is found to be under strong selection in the maize stiff-stalk group (Zhang et al., 2020). The aforementioned studies demonstrate that cloning and modulation of cell wall regulatory genes have contributed significantly to stem improvement in crops.

Stem anatomy is another key trait determining the stem mechanical strength. In rice, basal culm diameter, vascular bundle number, vascular bundle area, and vascular xylem area all significantly correlate with mechanical strength (Zhang et al., 2009). Also, multiple linear regression analysis shows that the majority variation of lodging resistance in maize is mainly dependent on the width of the mechanical tissue layer (Kong et al., 2013). Moreover, modifying the expression of genes related to stem structure development is proved to be applicable to improve stem strength. In stems, overexpression of *WOX14* (*WUSCHEL HOMEOBOX RELATED 14*) results in accumulation of bioactive GA, inducing strong lignification of secondary xylem (Denis et al., 2017). The Class II KNOTTED1-like homeobox (*KNOX2*) genes, *KNAT7* and *KNAT3*, act as a regulator of secondary cell wall biosynthesis in xylem vessels. Upregulation of *KNAT7* and *KNAT3* increases stem tensile and flexural strength (Wang et al., 2020). These worthwhile studies have significantly increased the theoretical underpinnings for increasing stem strength and yield in the corresponding crops.

In this study, we employed comparative biology and transcriptome analysis to identify the causes of stem lodging resistance in the star rapeseed variety ZS11. Our results showed that ZS11 has excellent phenotypes in SBS and RPR. Further physiological and anatomical analyses showed that ZS11 exhibited higher lignin and cellulose concentrations, thicker xylem cell layers, and more tightly arranged interfascicular fibrocytes compared with 4D122 (sensitivity of stem lodging). Comparative transcriptome analysis suggests that the differential expression of genes related to lignin and cellulose synthesis and modification pathways, as well as genes regulating secondary structural development of stem cell layers, might be responsible for the formation of high stem mechanical strength in ZS11. Finally, our results provide an insight into the efficient breeding application of ZS11 stem lodging resistance.

## Material and methods

### Plant materials and growth conditions

Rapeseed inbred lines of ZS11 and 4D122 were gown at the oil crops research institute WUCHANG experimental station (30°60′N, 114°30′E), of the Chinese academy of agricultural sciences. Seeds were sown on 28 September, 2020. Each field plot was 2.0 m×5.6 m with 16 rows (0.35 m between rows, with a planting density of 17000 plants per 667 m^2^).

### Measurement of lodging degree

Calculation of the lodging degree was conducted as previously described (Qiao, 1988; Tian and Yang, 2005) in each plot at maturity. Lodging degree of rapeseed was classified as grade 1 (upright) to grade 5 (fully prostrate) according to the angle between the main stem and the ground. A lower degree of lodging indicates greater resistance to lodging. For each inbred line, twenty plants were randomly selected for surveying, and the weighted average value was used to indicate the lodging degree.

### Measurement of mechanical properties of stem

To measure stem strength in rapeseed, six individual plants were randomly selected from each rapeseed line at flowering, and silique stages. In this study, 6 days after blooming was designated as flowering stage and 20 days after final flowering was considered the silique stage. The rind penetrometer resistance (RPR) of each internode, and the stem breaking force (BF) of the sixth and ten internodes from the top were measured with a YYD-1 stem strength tester (Top Cloud-Agri Technology Co., Zhejiang, China) according to a previous study (Shao et al., 2022). The stem breaking strength (SBS), D refers to the diameter of the internode corresponding to BF was calculated with the following formula: SBS = BF/(π × (D/2)^2^). Then, each internode was divided into three equal sections for measurement of cell wall components, anatomical characterization, and transcriptome analysis.

### Histochemical staining

According to the protocol established by (Li et al., 2011), the rapeseed stems of six internodes were selected at flowering and silique stages, cut into 1-2 cm sections and preserved in 70% FAA fixative. Afterwards, samples were dehydrated and transparentized with a concentration gradient of alcohol, xylene solution, and then embedded with paraffin wax. The embedded samples were sectioned with a Leica RM2016 slicer. Paraffin sections (4 µm) were dewaxed sequentially with different concentration gradients of alcohol and xylene (opposite to the dehydration and transparency steps). The slices were stained with 1% Safranin-O and 0.5% Fast Green solution, and dehydrated with absolute ethanol. Xylene was used to transparentize the samples for 5 min and sealed with neutral gum, then photographed under a light microscope (EX30, SOPTOP, China). Lightools software was used to measure the thickness of the sclerenchymatous hypodermis layer and the number and area of vascular bundles and interfascicular fibrocytes.

### Determination of cellulose and lignin concentrations

The remainder of the sixth internodes from the top of each line were sampled to determine lignin and cellulose concentrations. The samples were dried to a constant weight at 80°C. Samples were crushed into powder, then 20 mg powder was used for preparing alcohol-insoluble residues (AIRs) of the cell walls. Total lignin concentration was determined by the AcBr method (Iiyama and Wallis, 1990; Fukushima and Hatfield, 2004). According to the protocol established by (Updegraff, 1969), the 5 mg AIRs were hydrolyzed in Updegraff reagent (HNO_3_: HOAC: H_2_O,1:8:2) at 100°C for 30min. The concentrated samples were washed with acetone and hydrolyzed with sulfuric acid. The supernatant (dilute 20-fold) was used for measuring the cellulose amount by the anthrone assay. Firstly, different concentrations of glucose standard solutions (0.09, 0.08, 0.07, 0.05, 0.025, 0.0125, 0.00625 mg/mL) were prepared and used to plot the standard curves. Volumes of 630 µL of concentrated sulfuric acid and 70 µL of anthrone were added to 300 µL of the measured samples, standard solutions, and blank tubes and were mixed and then placed in a water-bath at 95°C for 10 min. After the reaction was completed and cooled to room temperature, the absorbance value at 620 nm was measured. The cellulose concentration (x, mg/mL) of the sample was calculated according to the plotted standard curve. The cellulose concentration (mg/g) was measured by (x × V × 20 × W2)/(W3 × W1 × 1.11), where V was volume of cellulose extraction solution, W1 was sample quality, W2 was AIRs quality, W3 was quality of AIRs weighed during cellulose extraction, 20 was the dilution ratio of the sample to be tested, and 1.11 was the constant for the conversion of glucose content to cellulose concentration (i. e. 111 µg of glucose color developed with anthrone reagent was equivalent to the color shown by 100 µg of cellulose anthrone reagent).

### Measurement of SAM and Met concentrations and exogenously applied SAM treatment

The stems of the ZS11 and 4D122 plants at the flowering and silique stages were collected to determine the concentration of S-adenosylmethionine (SAM) and methionine (Met). Approximately 50 mg of freshly collected stem sample was ground in PBS solution (0.01 M, pH = 7.4). After centrifugation at 5000 g for 15 min, the supernatants were immediately used to measure SAM and Met concentrations by ELISA kits (Kit number: ml034411 for SAM measurement, ml034795 for Met measurement; Shanghai Enzyme-linked Biotechnology Co., Ltd., China), according to (Bai et al., 2018).

Exogenous application of SAM treatment was performed in the greenhouse. Two SAM solution concentration gradients of 100 and 1000 μM were installed and the leaves were sprayed in equal amounts at the bolting stage. Untreated 4D122 and ZS11 were used as controls The RPR was measured by the sixth internode of the stem at the flowering stage. And then the SAM concentration was determined by the previous method (Bai et al., 2018). At least five replicates of each treatment were obtained.

### RNA-seq analysis

Samples of the sixth internodes of ZS11 and 4D122 stems at the flowering and silique stages were used for RNA-seq analysis. Stem tissue samples were collected from 3 representative plants per line.

Total RNA extraction, RNA-seq library construction and sequencing were carried out at Beijing Novogene Bioinformatics Technology Co. (Beijing, China). A NEBNext^®^ Ultra™ Directional RNA Library Prep Kit for Illumina^®^ (NEB, MA, USA) was used for RNA-seq library construction. The RNA quality and integrity were assessed using Aligent 2100. After passing the library inspection, different libraries were pooled according to the effective concentration and target downstream data volume were sequenced with the Illumina Hiseq 2500 platform.

The low qualitative reads in original raw reads were excluded using fastp preprocessor (Chen et al., 2018). Reads that passed the filter were then aligned to the rapeseed ZS11.v0 reference genome (http://yanglab.hzau.edu.cn/BnIR/germplasm_info?id=ZS11.v0) using HISAT2 v 2.0.5 (Kim et al., 2015). The number of reads mapped to each gene were counted using featureCounts (1.5.0-p3) to quantify the gene expression levels (Yang et al., 2014). Only perfectly matching sequences were considered for further analysis. The normalized gene expression levels were determined with count information as FPKM (Fragments Per Kilobase of transcript per Million mapped reads). Expressed genes with FPKM ≥ 1 were used for the comparative analysis (Xie et al., 2022). Differential expression analysis was performed using the DESeq2 R package (v1.16.1) (Love et al., 2014). Genes were considered as DEGs if the |log_2_(FoldChange)| ≥1 and *padj* ≤ 0.05. Subsequently, we manually removed low expression abundance DEGs, based on at least two replicates in ZS11 or 4D122 with FPKM = 0. Gene ontology (GO) and KEGG enrichment analyses were performed using clusterProfiler (v3.4.4) with a corrected *p*-value ≤ 0.05 (Young et al., 2010).

### Protein interaction network analysis

Firstly, the DEGs associated with secondary cell wall synthesis and vascular development were screened by analyzing the gene enrichment results as well as information reported in the literature. Secondly, the corresponding Darmor gene ID in the BnIR database (http://yanglab.hzau.edu.cn/BnIR/jbrowse) were searched, and then the String database (https://cn.stringdb.org/cgi/input?sessionId=bP7h2SXS6Qr5&input_page_active_form=multiple_identifiers) was used to search for the node information of the related protein encoded by the corresponding gene. Finally, the protein interaction network was constructed of Cytoscape software (v3.8.0). Transcription factors and structural proteins involved in vascular development were retained and cell wall component-related proteins were deleted. The nodes were arranged according to betweenness centrality. Larger values indicate greater influence of the nodes in the network. The size of the font is related to the betweenness centrality, with larger fonts accompanied by larger betweenness values. In the case of Node Fill Color Mapping by Log2FoldChange at flowering stage, negative values indicate down-regulation, while positive values indicate up-regulation. Meanwhile, up-regulated and down-regulated expression of the protein showed different colors.

### Statistical analyses

One-way ANOVA and Duncan’s *post hoc* test were conducted using SPSS 19.0 statistical software (SPSS, Inc., IL, USA). *p*-value ≤ 0.05 was considered statistically significant. Standard errors are provided in all figures and tables were appropriate.

## Results

### ZS11 exhibited significant advantages in stem structure

**Figure 1:**
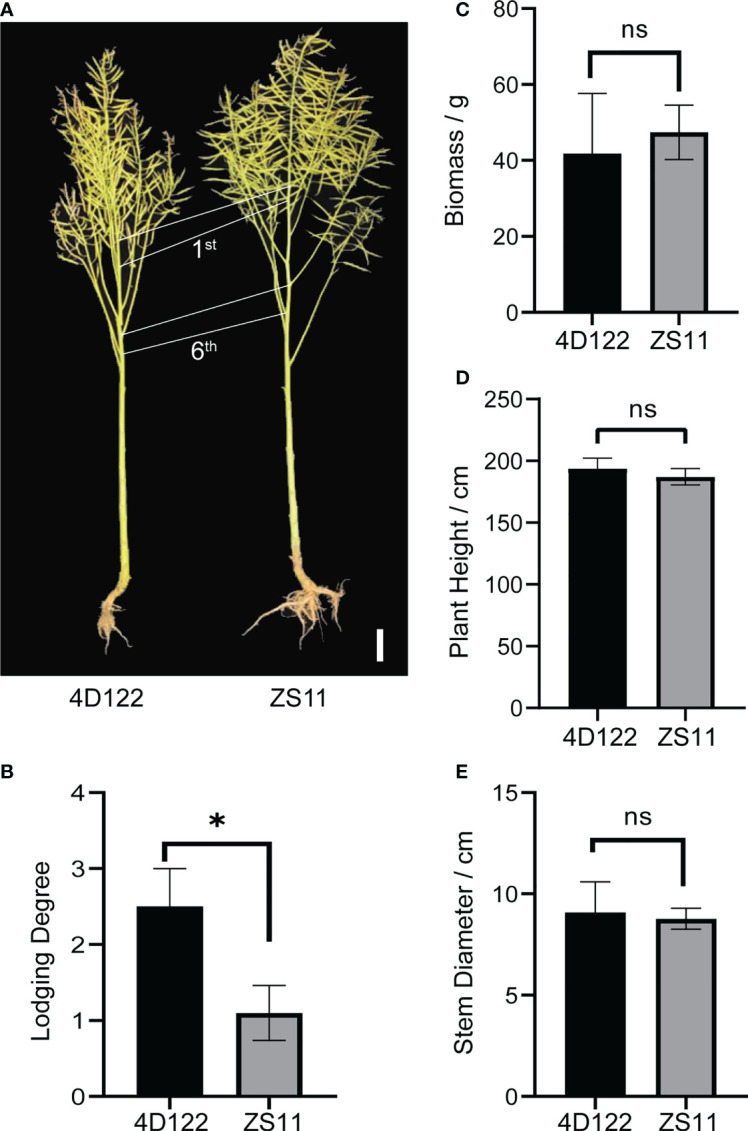
Characterization for plant types and stem lodging traits of ZS11 and 4D122. **(A)** Image of ZS11 and 4D122 plants, 1^st^: the first internode, 6^th^: the sixth internode. **(B–E)** Lodging Degree, Biomass, Plant Height, and Stem Diameter between 4D122 and ZS11 at silique stage. Error bars represent standard deviation (n = 8). ns refer to not significant with *p*-value > 0.05 by t-test. * refer to not significant and *p*-value ≤ 0.05 by *t*-test, respectively.

ZS11 is a widely planted semi-winter rapeseed variety with outstanding lodging resistance for both root and stem. In order to explore the specific lodging-resistant advantages of ZS11 in aerial regions, we selected a lodging material 4D122 for comparative biology research. As shown in **Figures 1A, B**, ZS11 showed no clear difference in plant type traits such as plant height compared with 4D122, but had a much lower lodging degree. Furthermore, 4D122 plants showed severe stem breakage in field conditions covered with nets (**Supplementary Figure S1**). We evaluated several lodging related traits, including aerial biomass, plant height, and stem diameter at silique stage. No significant differences were detected for these three traits between ZS11 and 4D122 (**Figures 1C–E**; **Supplementary Table S1**). These results suggested that the difference in stem lodging resistance between these two materials is not the resulted of plant architecture.

**Figure 2:**
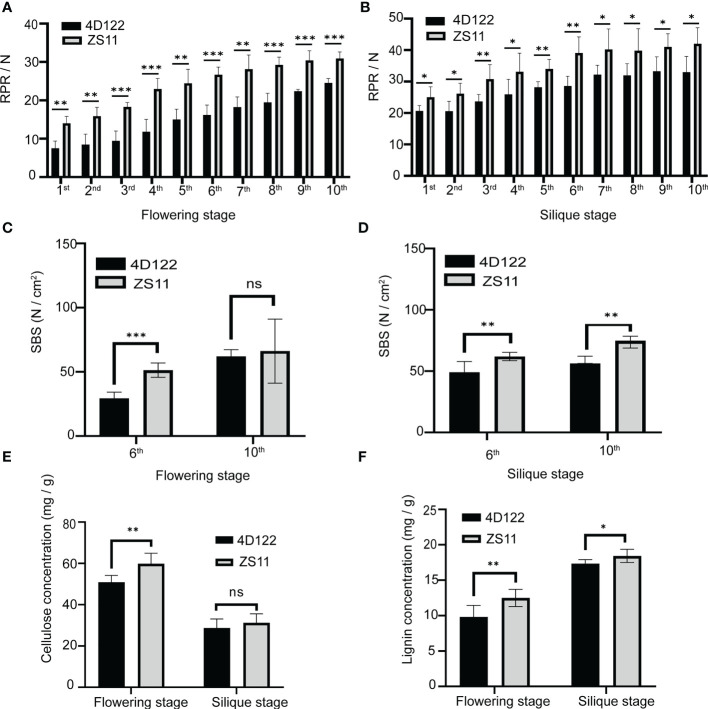
Characterization of rind penetrometer resistance (RPR), stem breaking strength (SBS), and stem chemistry composition of ZS11 and 4D122 at flowering and silique stages. **(A, B)** RPR between ZS11 and 4D122 in each internode at flowering and silique stages. **(C, D)** SBS of ZS11 and 4D122 in the 6^th^ and 10^th^ internodes. **(E, F)** Cellulose and lignin concentrations in the 6^th^ internode. Error bars represent standard deviation (n = 6). ns refer to not significant with p-value > 0.05 by *t*-test. *, **, and *** refer to *p*-value ≤ 0.05, *p*-value ≤ 0.01 and *p*-value ≤ 0.001 by *t*-test, respectively.

Then we investigated the rind penetrometer resistance (RPR) and stem breaking strength (SBS) of different internodes to characterize the differences in stem mechanical strength between ZS11 and 4D122. At flowering stage, the RPR values of the 1^st^ internode to the 10^th^ internode gradually increased from 14.0 ± 1.8 N to 30.9 ± 1.7 N in ZS11. In 4D122, the RPR values varied from 7.5 ± 1.9 N to 24.5 ± 1.1 N (**Supplementary Table S2**). Further statistical analysis showed that the RPR values of ZS11 internodes were significantly higher than those of 4D122 with the same node position (**Figure 2A**; **Supplementary Table S2**). At silique stage, the RPR values of measured internodes varied from 25.0 ± 3.3 N to 42.0 ± 5.1 N in ZS11 and from 20.6 ± 1.7 N to 33.0 ± 5.0 N in 4D122 (**Figure 2B**; **Supplementary Table S2**). A significantly higher RPR value of each ZS11 internode was also detected at silique stage (**Figure 2B**). T-test analysis revealed that the most striking RPR differences were detected in the 6^th^ internode at both stages (*p*-value^flowering stage^ = 1.5E-05, *p*-value^silique stage^ = 1.6E-03) (**Figures 2A, B**; **Supplementary Table S2**).

Subsequently, we calculated the SBS values of the internodes 6 and 10. At flowering stage, the SBS values of the 6^th^ and 10^th^ internodes of 4D122 were 29.5 ± 4.7 N and 57.3 ± 5.1 N, respectively, which were obviously lower than those of ZS11 (51.3 ± 5.5 N and 62.2 ± 11.5 N) (**Figure 2C**; **Supplementary Table S3**). At silique stage, the SBS values of the 6^th^ and 10^th^ internodes in both 4D122 and ZS11 were 49.1 ± 8.7, 56.2 ± 5.8, 62.0 ± 3.4, 66.4± 4.8 N, respectively (**Figure 2D**; **Supplementary Table S3**). These results show that the SBS of internodes in both parents only increased slightly from flowering to silique stage. Additionally, the SBS values of the 6^th^ internodes in ZS11 were always significantly higher than those in 4D122 (**Figures 2C, D**). Therefore, it is reasonable to speculate that excellent stem mechanical strength is the main reason of strong stem lodging resistance in ZS11.

### Comparative analysis of cell wall composition

The mechanical strength of stem is mainly determined by cell wall composition and anatomical structure of stem tissues (Zhang et al., 2020). We first investigated the concentrations of cellulose and lignin, the two components that contribute the most to stem mechanical strength. AIRs of the 6^th^ internode were used for analysis. As shown in **Figure 2E**, the cellulose concentrations of ZS11 (60 ± 5 mg/g) internodes were significantly higher than those of 4D122 (51 ± 3 mg/g) at flowering stage (**Supplementary Table S4**). Compared to flowering stage, the internode cellulose concentrations of ZS11 and 4D122 were reduced by 48% and 44% at silique stage. The results of statistical analysis indicated that there was no significant difference in cellulose concentration between these two cultivars at silique stage (*p*-value = 0.34) (**Figure 2E** and **Supplementary Table S4**).

From flowering stage to silique stage, internode lignin concentration increased from 10 ± 2 to 17.4 ± 0.6 mg/g in 4D122 and from 13 ± 1 to 18.4 ± 0.9 mg/g in ZS11 (**Figure 2F**; **Supplementary Table S4**). At both developmental stages, internode lignin concentration of ZS11 was always higher than that of 4D122. The difference in lignin concentration between these two materials was much greater at the flowering stage than at the silique stage (**Figure 2F**). These results demonstrated that ZS11 can accumulate more lignin than 4D122 to enhance the mechanical strength of the stem.

### Characterization of internode anatomical architecture

**Figure 3:**
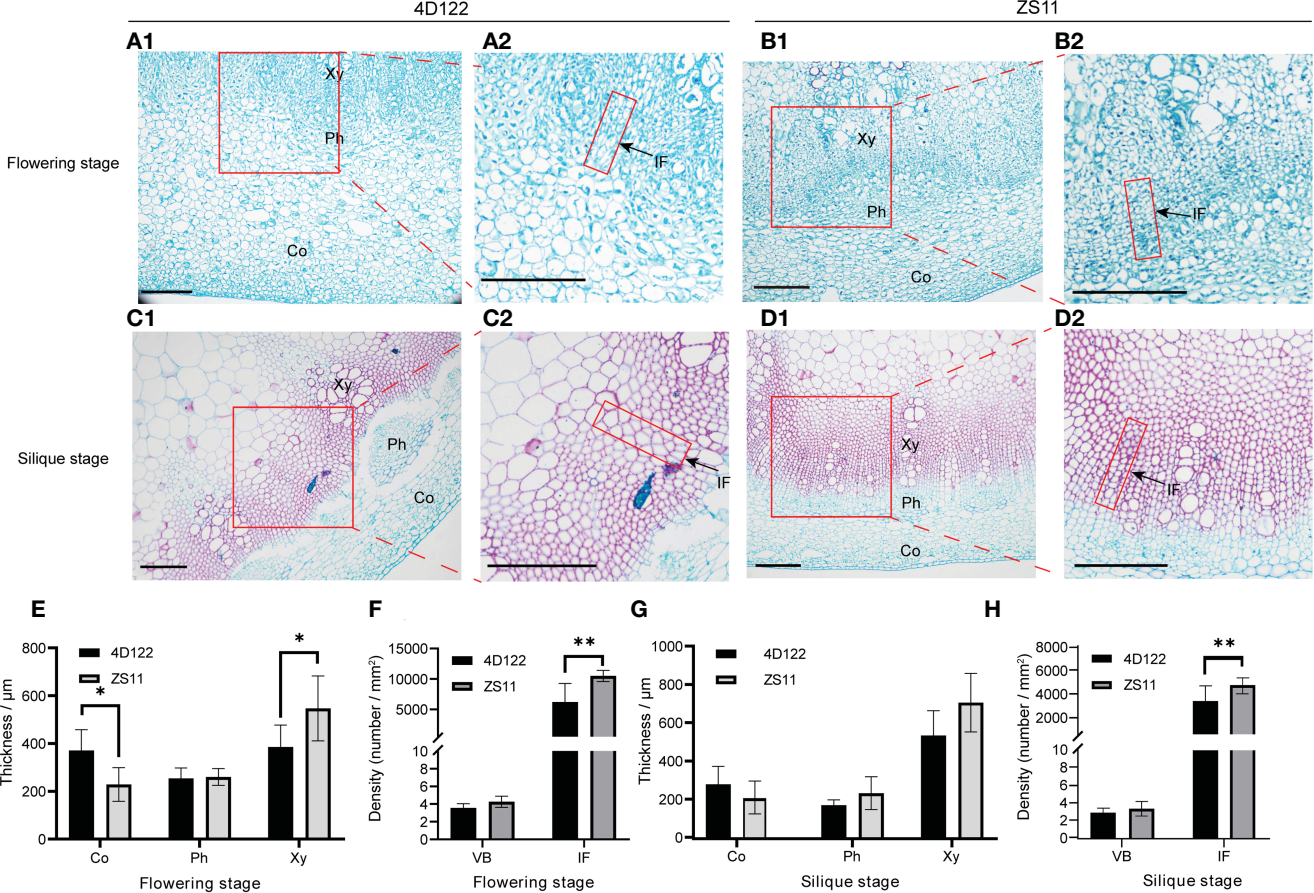
Microstructure of the sixth internode of ZS11 and 4D122. **(A–D)** Images of transection of the sixth internode. A1, B1, C1, D1 are magnified by 10 times, Scale bars = 200 μm; A2, B2, C2, D2 are equal-scale magnification of A1, B1, C1, D1. **(E–H)** Anatomical characteristics of Cortex (Co) thickness, phloem (Ph) thickness, xylem (Xy) thickness, interfascicular fibrocytes density (IF), and vascular bundle (VB) density were measured at flowering and silique stages. Error bars represent the standard deviation (n = 6), * and ** refer to *p*-value ≤ 0.05 and *p*-value ≤ 0.01 by *t*-test, respectively.

In order to uncover the differences in internode anatomical structure between ZS11 and 4D122, we performed cross-section analysis of the sixth internode at flowering and silique stages. We used saffron-solid green staining analysis to distinguish tissues’ lignification. At flowering stage, the cell wall of cortex, phloem, and xylem were less lignified (**Figures 3A1, B1**). At silique stage, the lignification extent of cell wall was higher in the xylem, while it was relatively lower in the cortex and phloem (**Figures 3C1, D1**). We then measured the thickness of the cortex, phloem and xylem. At both investigated stages, the phloem thickness of the 6^th^ internode in ZS11 was about the same as that in 4D122 (**Figures 3E, G**). The cortex thickness of ZS11 internode was consistently lower than that in 4D122, and only at the flowering period did the difference reach a significant level (**Figures 3E, G**). From flowering stage to silique stage, the xylem thickness increased from 385.2 ± 91.2 to 532.5 ± 130.3 μm in 4D122, and from 546.7 ± 136.0 to 704.7 ± 153.0 μm in ZS11 (**Supplementary Table S5**). By statistical analysis, the results showed that the xylem thickness of ZS11 was significantly higher than that of 4D122 at flowering stage (**Figure 3E**).

To more distinctly characterize the difference of vascular structure, we further selected vascular bundle density and interfascicular fibrocyte density (the number of interfascicular fibrocyte per unit area) to evaluate the tissue tightness of the lignified area (**Figures 3A2–D2**). For the trait vascular bundle density, we observed no obvious differences between these two varieties (**Figures 3F, H**). Compared to 4D122, the density of interfascicular fibrocytes of the ZS11 internode was 109.3% higher at flowering stage (**Figure 3F**; **Supplementary Table S5**) and 47.6% higher at silique stage (**Figure 3H**; **Supplementary Table S5**). Therefore, these cytological observations suggest that ZS11 indeed had a higher degree of secondary lignification of stem vascular tissue and also more closely distributed lignified cells.

### Comparative transcriptome analysis of internodes of ZS11 and 4D122

**Figure 4:**
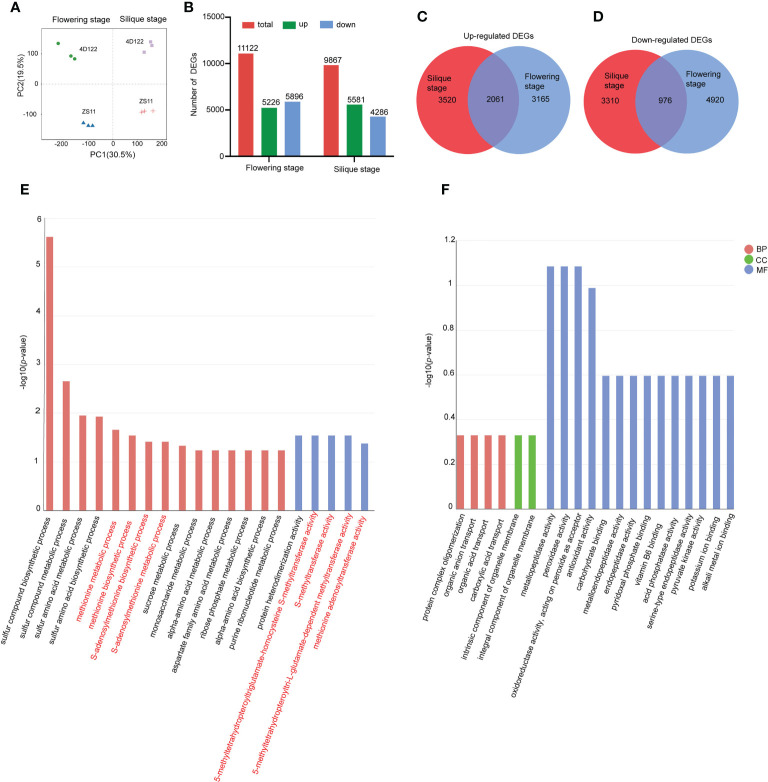
Transcriptome analysis in two rapeseed lines at flowering and silique stages. **(A)** Principal component analysis (PCA) of all four samples. **(B)** Number of total (total), up-regulated (up), and down-regulated (down) DEGs for ZS11 at the flowering and silique stages, compared with 4D122. **(C, D)** Venn diagram of up-regulated and down-regulated DEGs at two stages. **(E)** Top 20 significantly enriched Gene Ontology (GO) terms (*p-*value < 0.05) of up-regulated DEGs at the two stages. **(F)** Top 20 significantly enriched Gene Ontology (GO) terms (*p-*value < 0.05) of down-regulated DEGs at the two stages. The vertical coordinates of E and F are graphed as the log_10_^(^ *^p-^* ^value)^. BP: “Biological Process” subgroup, CC: “anatomical component” subgroup, MF: “Molecular Function” subgroup.

To explore the molecular mechanisms underlying the strong lodging-resistant property of ZS11 stems, the sixth internodes of ZS11 and 4D122 were collected at flowering stage and silique stage for comparative transcriptome analysis. After removing the low-quality sequences and adapter sequences, approximately 39.2 to 50.2 million clean reads were obtained (**Supplementary Table S6**). In each library, 80.8 to 93.3% of clean reads were uniquely mapped reads (**Supplementary Table S6**). Principal component analysis (PCA) showed that the three biological replicates of each material clustered together, which suggested that the transcriptome datasets were satisfactory (**Figure 4A**). The first and second principal components were able to discriminate samples with different developmental stages and different genetic backgrounds, respectively (**Figure 4A**).

To identify differentially expressed genes (DEGs), |log2(FoldChange)| ≥ 1 and *padj* ≤ 0.05 were used as filter thresholds. After removing genes with low expression levels, 11122 and 9867 DEGs between ZS11 and 4D122 were identified at flowering stage and silique stage, respectively (**Figure 4B**). Compared with 4D122, 5226 DEGs were significantly up-regulated and 5896 DEGs were significantly down-regulated in ZS11 at flowering stage. At silique stage, 5581 significantly up-regulated DEGs were detected, and 4286 DEGs showed significantly down-regulated expression levels (**Figure 4B**). Venn analysis showed that 2061 upregulated DEGs and 976 downregulated DEGs were consistently detected at both developmental stages (**Figures 4C, D**).

### GO annotation analysis and KEGG enrichment analysis of consistent DEGs

A GO annotation analysis was performed to identify differentially regulated function modules using the consistent DEGs identified above. In regards to the co-upregulated DEGs in ZS11, the top 20 significantly enriched GO terms were categorized into Biological Process (BP) and Molecular Function (MF) groups. These enriched items were mainly involved in sulfur compound biosynthetic and metabolic processes, methionine biosynthetic and metabolic processes, sucrose metabolic process, and monosaccharide metabolic process. The most significantly enriched MFs were 5-methyltetrahydropteroyltriglutamate-homocysteine S-methyltransferase activity, S-methyltransferase activity, 5-methyltetrahydropteroyltri-L-glutamate-dependent methyltransferase activity, and methionine adenosyltransferase activity (**Figure 4E**; **Supplementary Tables S7**, **S8**). GO enrich analysis of co-downregulated DEGs in ZS11 showed that mostly enriched items were annotated in the MF group, mainly containing metallopeptidase activity and peroxidase activity (**Figure 4F**; **Supplementary Tables S9**, **S10**).

KEGG enrichment analysis was employed to identify differentially utilized metabolic pathways by employing the consistent DEGs identified above. In regards to the co-upregulated DEGs in ZS11, the top 20 enriched KEGG terms were mainly involved in alpha-Linolenic acid metabolism, biosynthesis of unsaturated fatty acids, sulfur metabolism, and peroxisome (**Supplementary Figure S2A**). KEGG enrich analysis using co-downregulated DEGs in ZS11 showed that a majority of the top 20 pathways were enriched in the synthesis and metabolic pathways of various amino acids, such as arginine, proline, cysteine, and tyrosine (**Supplementary Figure S2B**).

### ZS11 exhibited a higher biosynthesis ability for Met and SAM than 4D122 during stem development

**Figure 5:**
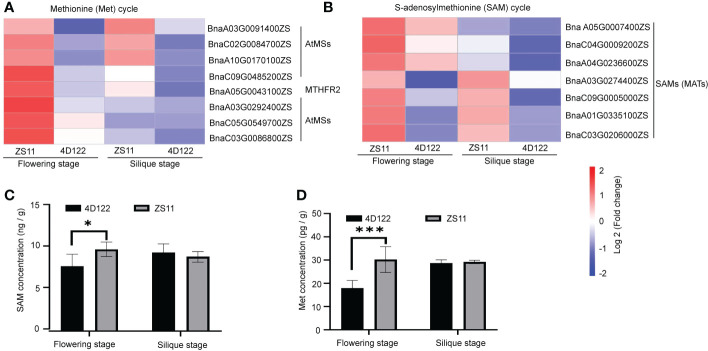
SAM and Met cycle related DEGs and concentrations in ZS11 and 4D122 at flowering and silique stages. **(A)** Differentially expressed genes associated with the Met cycle at two stages in ZS11 and 4D122. **(B)** Differentially expressed genes associated with the SAM cycle. **(C)** SAM concentration of ZS11 and 4D122. **(D)** Met content of ZS11 and 4D122. Error bars represent the standard deviation (n = 6), * and *** refer to *p*-value ≤ 0.05 and *p*-value ≤ 0.001 by *t*-test, respectively.

S-adenosylmethionine (SAM) is the activated form of methionine (Met), which is involved in the synthesis and modification of many metabolites (Sekula et al., 2020). In the lignin biosynthetic pathway, caffeoyl CoA Omethyltransferase (CCoAOMT) and caffeic acid O-methyltransferase (COMT) utilize SAM as a cofactor and ultimately lead to the biosynthesis of lignin monomers (Dixon and Barros, 2019). However, the down-regulated expression of DEGs was not enriched for a distinctly associated pathway (related to regulation of cell wall components, vascular bundle structure, etc.) (**Figure 4F**). In the co-enriched subgroups of up-regulated DEGs, 7 DEGs related from the *METS* (*MS*) family, which encode cytosolic cobalamin-independent methionine synthase, were involved in synthesis of Met from Hcy (homocysteine) (**Figure 5A**). One DEG, *MTHFR2* (*METHYLENETETRAHYDROFOLATE REDUCTASE 2*), was speculated to significantly affect lignin biosynthesis. Of these Met cycle-related DEGs, only five genes were from the *MS* family and *SAM* (*MAT*) family at silique stage, respectively, and expressions were significantly lower than those at flowering stage (**Figures 5A, B**). The levels of differentially expressed genes related to SAM metabolism were significantly higher at flowering stage than at silique stage (**Figures 5A, B**).

The concentrations of SAM and Met showed the same pattern of variation as the above gene expression. Among the top 20 categories of GO annotation analysis, many SAM- and Met- related process groups might be involved in etrahydrofolate (THF) and Met cycles at one-carbon (C1) metabolism by associating with biosynthesis of methionine and S-adenosylmethionine. The results of ELISA analysis of SAM and Met showed that ZS11 contained 21% higher SAM concentration than 4D122 at flowering stage (**Figures 5C, D**). Meanwhile, the Met concentration increased by 41% in ZS11 at flowering stage (**Figure 5D** and **Supplementary Table S11**).

To further demonstrate the role of SAM on the formation of stem mechanical strength, we used different concentrations of exogenous SAM (100 and 1000 μM) to treat 4D122 plants at bolting stage and then measured the RPR values of the sixth internode at flowering stage. Compared to untreated group, 100 and 1000 μM treatments significantly increased the concentration of SAM and also the corresponding RPR values (**Supplementary Figure S3**). These results showed that exogenous SAM treatment enhanced the SAM concentration, leading to an increase in the RPR values of the stems, implying that the high SAM concentration may be one of the reasons for the superiority stems in ZS11.

### Identification of DEGs involved in cellulose synthesis

**Figure 6:**
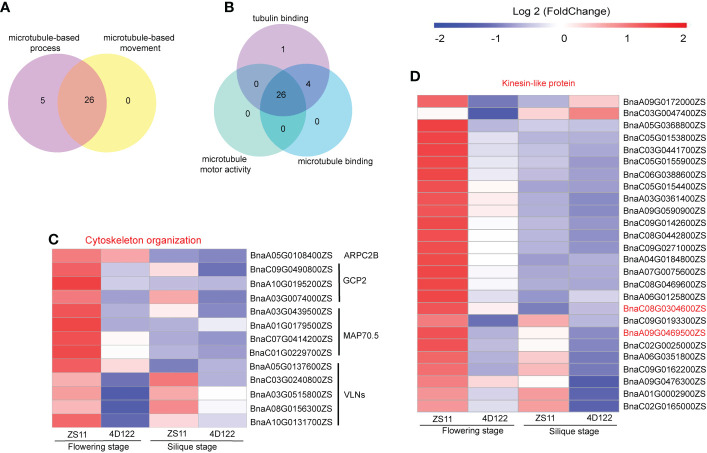
Clustering Chart of DEGs about cellulose synthesis of ZS11 and 4D122 at flowering and silique stages. **(A)** Venn diagram of microtubule-based process and microtubule-based movement of biological process group (BP) at flowering stage. **(B)** Venn diagram of tubulin binding, microtubule binding, and microtubule motor activity of molecular function group (MF) at flowering stage. **(C)** DEGs involved in cytoskeleton organization at two stages. **(D)** DEGs encoded kinesin-like proteins of five microtubule related process at two stages.

The above phenotypic analysis results showed that there were significant differences in cellulose and lignin concentrations between ZS11 and 4D122. Therefore, further analysis of genes related to cellulose and lignin synthesis in the GO analysis results was performed. In the significantly enriched GO terms of up-regulated DEGs at flowering stage, microtubule-related processes were significantly enriched. For example, microtubule-based process, microtubule-based movement in biological process group, tubulin binding, microtubule binding, and microtubule motor activity in molecular function group were significantly enriched (**Supplementary Table S7**). A total of 26 DEGs were clustered into these microtubule-based processes and microtubule-based movement subgroups (**Figure 6A**). Similarly, there were 26 DEGs clustered into tubulin binding, microtubule binding, and the microtubule motor activity of molecular function group (**Figure 6B**). All of these 26 DEGs encoded Kinesin-like proteins (**Figure 6D**). Among these proteins, *BnaC08G0304600ZS* and *BnaA09G0469500ZS* were of the kinesin-4 family. Kinesin-4 is involved in cortical microtubule transport of non-cellulosic components during cell elongation in relation to cell wall mechanisms in *Arabidopsis thaliana* (Kong et al., 2015). We therefore speculated that microtubule processes might affect transport of cell wall synthetic materials and the content of cell wall components.

The cytoskeleton is an important component for maintaining anatomical morphology, consisting of microfilaments and microtubules, as well as being involved in a wide range of anatomical motilities (Zang et al., 2021). In this study, 13 DEGs for cytoskeleton organization were significant up-regulated at flowering stage (**Figure 6C**), and *MAP70.5* and *GCP2* were additionally involved in microtubule-related regulation (Pesquet et al., 2010; Zhou and Li, 2015). The remaining DEGs were actin-related proteins., such as *AtMAP70-5*, which regulates cellulose synthase motility and secondary wall patterning (Pesquet et al., 2010; Xiao et al., 2016).

### Identification of DEGs involved in lignin synthesis

**Figure 7:**
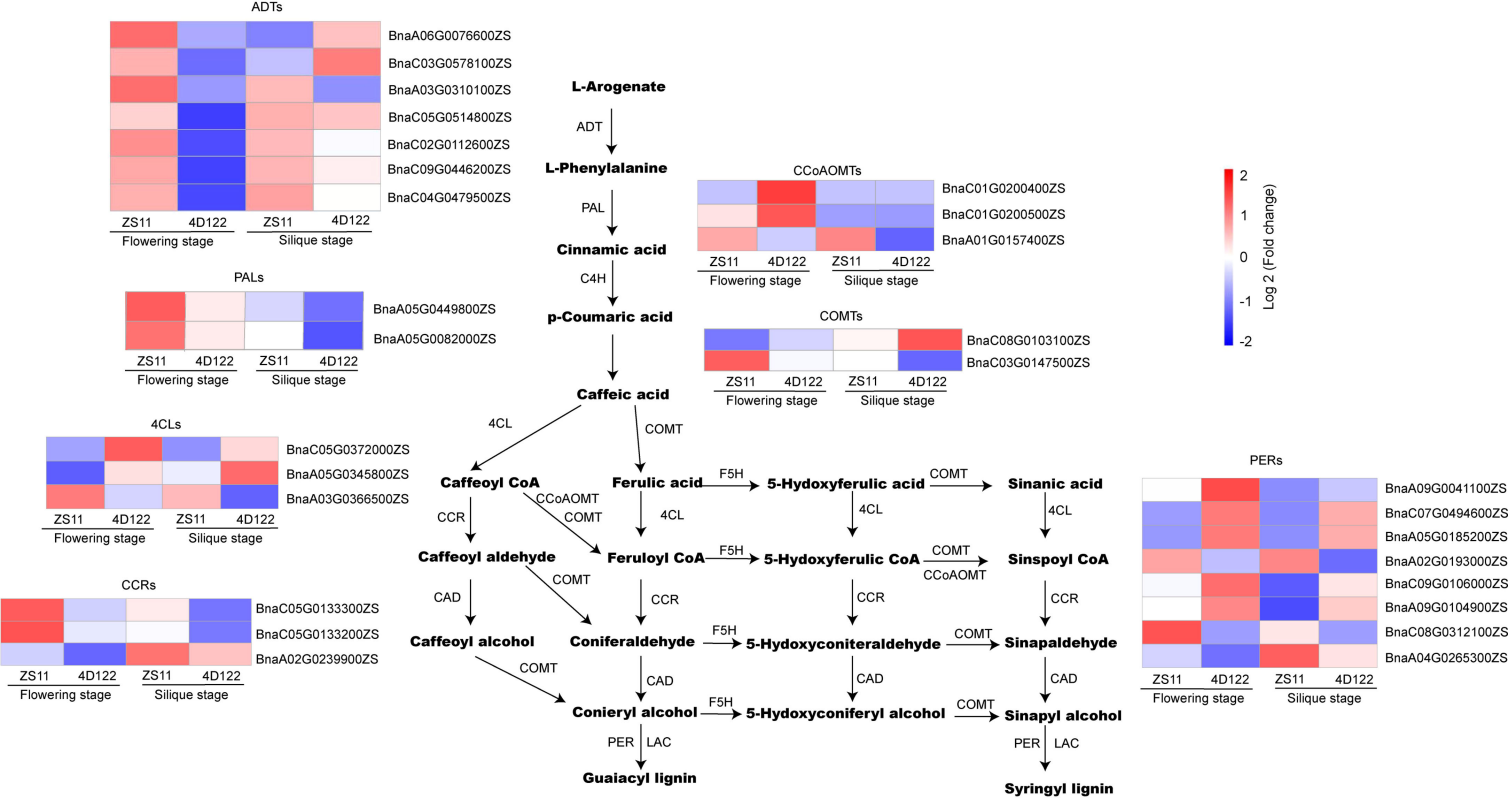
Identification of DEGs involved in lignin synthesis at flowering and silique stages. ADT: arogenate dehydratase, PAL: Phenylalanine ammonia-lyase, C4H: Cinnamate 4-Hydroxylase, 4CL: 4-coumarate-CoA ligase, CCR: Cinnamoyl-CoA reductase, CCoAOMT: caffeoyl-coenzyme A O-methyltransferase, COMT: Caffeic acid 3-O-methyltransferase, CAD: Cinnamyl alcohol deaminase, F5H: Ferulate 5-hydroxylase, LAC: LACCASE, PER: Peroxidase.

Significant differences in lignin concentration were observed at flowering and silique stages, which were described above. Therefore, we analyzed DEGs related to the lignin metabolic pathway. GO analysis showed that the L-phenylalanine biosynthetic process and the L-phenylalanine metabolic process were enriched at flowering stage (**Supplementary Table S7**). These two metabolic processes each involved in 7 DEGs (*BnaC02G0112600ZS*, *BnaA06G0076600ZS*, *BnaA03G0310100ZS*, *BnaC03G0578100ZS*, *BnaC09G0446200ZS*, *BnaC04G0479500ZS*, *BnaC05G0514800ZS*) and encoded plastid-localized arogenate dehydratase (ADTs) (**Figure 7**). In *Arabidopsis thaliana*, ADTs were hypothesized to differentially control carbon flux for lignin deposition (Corea et al., 2012). On the other hand, the homologous genes of the lignin metabolic pathway were not always robustly upregulated in ZS11 (**Figure 7**). For example, only *BnaA03G0366500ZS* (*4CL4*), *BnaA01G0157400ZS* (*CCoAOMT*), and *BnaC03G0147500ZS* (*OMT1*) were significantly upregulated DEGs in the clustering diagram (**Figure 7**). In the clustering chart of *CCRs* and *PERs*, 4 genes (*BnaC05G0133300ZS* and B*naC05G0133200ZS* in *CCRs* and *BnaA02G0193000ZS* and *BnaC08G0312100ZS* in *PERs*) showed a significant up-regulation trend at flowering stage (**Figure 7**). Therefore, we speculated that the up-regulated expression of multiple regulatory genes in the lignin metabolism pathway ultimately resulted in enhanced lignin concentration of stem internodes in ZS11.

### Protein interaction network analysis of DEGs associated with vascular development

**Figure 8:**
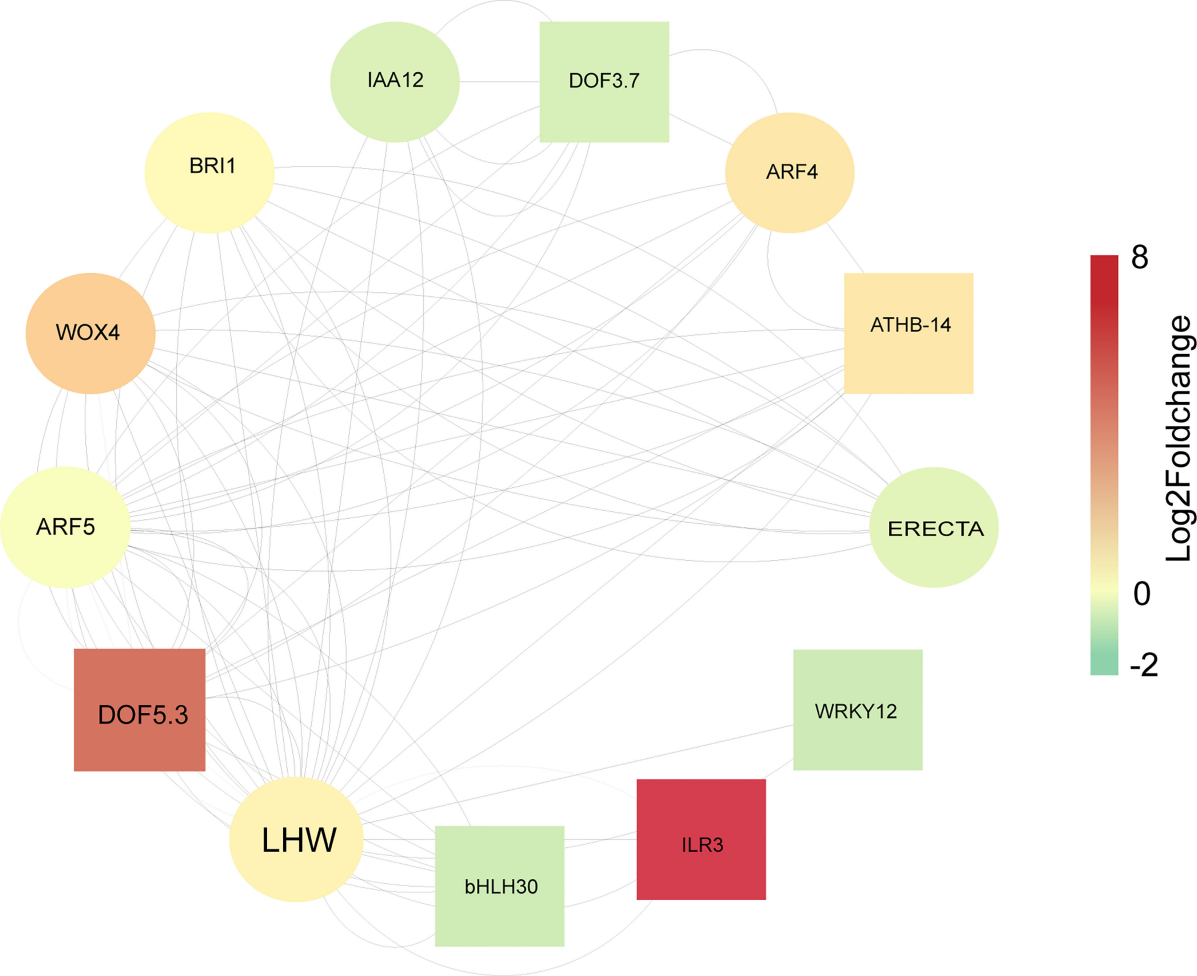
Diagrammatic Representation of proteins encoded by differentially expressed genes related to vascular development at flowering or silique stages. The nodes are arranged according to betweenness centrality. The size of the font is related to the betweenness centrality, with larger fonts accompanied by larger betweenness values. Larger values indicate greater influence of the nodes in the network. Node Fill Color Mapping by Log2FoldChange at flowering stage, negative values indicate down-regulated, positive values indicate up-regulated. Square nodes are transcriptional regulators, Circular nodes are Vascular bundle development-related proteins. According to the main using regions of these proteins corresponding to the encoded genes, we divided them into four categories. The first category includes BRI1(BRASSINOSTEROID INSENSITIVE), ATHB-14 (ARABIDOPSIS THALIANA HOMEOBOX PROTEIN 1), IAA12 (Auxin/Indole-3-Acetic-Acid12), DOF5.3 (DNA BINDING WITH ONE FINGER 5.3), DOF3.2, ARF4 (AUXIN RESPONSE FACTOR 4), ARF5, and WOX4 (WUSCHEL HOMEOBOX RELATED 4), which mainly act on the cambium. The second category includes LHW (LONESOME HIGHWAY), bHLH30 (BASIC HELIX-LOOP-HELIX 30), which mainly act on the xylem. The third one includes ERECTA (LEUCINE-RICH REPEAT RECEPTOR-LIKE SER/THR KINASE), which is mainly acts on the phloem. The last one includes WRKY12 (WRKY DNA-BINDING PROTEIN 12) and ILR3 (IAA-LEUCINE RESISTANT3), with no obvious acting region.

To investigate the relationship of DEGs related to secondary cell wall synthesis and vascular development, a total of 1029 genes associated with vascular bundle structure and cell wall development were first screened by reviewing the literature and related databases, of which 322 genes were differentially expressed in the sixth internode of the stem at the flowering or silique stages (**Supplementary Figure S4**). We compared known vascular development-related genes with the consistent DEGs described above, and then used cytoscape software to construct an interactive regulatory network consisting of 13 proteins (**Supplementary Tables S12**-**S14**). According to the protein functions, these proteins are mainly involved in transcriptional regulation such as DOF5.3 (DNA BINDING WITH ONE FINGER 5.3), DOF3.7, ATHB-14 (ARABIDOPSIS THALIANA HOMEOBOX PROTEIN 1), WRKY12 (WRKY DNA-BINDING PROTEIN 12), ILR3 (IAA-LEUCINE RESISTANT3), and bHLH30 (BASIC HELIX-LOOP-HELIX 30*)* (the square nodes in **Figure 8**), or acted as structural proteins to regulate vascular bundle development such as LHW (LONESOME HIGHWAY), ARF5 (AUXIN RESPONSE FACTOR 5), WOX4 (WUSCHEL HOMEOBOX RELATED 4), BRI1 (BRASSINOSTEROID INSENSITIVE 1), and ARF4 (the circular nodes in **Figure 8**). LHW is the node with the highest betweenness value, followed by DOF5.3, ARF5, WOX4, and BRI1. Furthermore, all corresponding encoding genes were up-regulated except BRI (**Figure 8**). *LHW* is a key regulator that initiates vascular cell differentiation (Ohashi-Ito et al., 2019). The Log2(FoldChange) values indicate that the most significantly up-regulated expression was *ILR3*, followed by *DOF5.3*. In contrast, the highest down-regulated expression was *WRKY12*, accompanied by *bHLH30* (**Figure 8**; **Supplementary Table S14**). These genes positively regulate vascular development, and they are both directly or indirectly regulated by signaling of auxin, ABA, or GA (Ranocha et al., 2013; Yang et al., 2016; Ohashi-Ito et al., 2019). Meanwhile, the stem strength of *Brassica napus* is complex, constituting from a substantial regulatory network with dynamic expression of those genes.

## Discussion

Lodging impacts rapeseed yield accumulation and complicates mechanized harvesting. Stem lodging is one of the main lodging types, which is inseparable from the stem’s mechanical properties and structures (Kuai et al., 2016; Weng et al., 2017). In this study, we compared ZS11 with 4D122 (lower stem strength) in terms of stem mechanical, anatomical, and biochemical traits and performed a comparative transcriptome analysis to understand the formation of ZS11’s superior stem strength.

### ZS11 possesses a denser lignified cell layer

A typical dicotyledonous plant stem is mainly composed of epidermis, cortex and vascular bundles. The cortex and vascular bundles are the main support structures that contain much of the thick sclerenchyma (Wang, 2020). In the present study, we showed that ZS11 exhibited a thicker xylem (**Figure 3**). Histochemical staining also revealed that the xylem of ZS11 was thicker than that of 4D122 (**Figures 3A–D**). Further statistical measurement analysis showed the interfascicular fibrocytes of ZS11 were more closely arranged (**Figures 3E–H**). We introduced the parameter of interfascicular fibrocyte density to more clearly characterize this difference. The results showed that ZS11 contained more interfascicular fibrocytes in the same area compared to those of 4D122.

Transcriptome analysis revealed that multiple genes (such as *LHW*, *DOF5.3*, *ARF5*, *ARF4*, *WOX4*) regulating vascular development had higher expression in ZS11 compared to those in 4D122 (**Figure 8**). *LHW* is required for promoting the expression of *MP* (*ARF5*) and *ATHB-8* in the provascular region (Ohashi-Ito et al., 2013), and *ARF4* regulates shoot regeneration through competing with *ARF5* for interaction with *IAA12* (Zhang et al., 2021). The heterodimer complexes of LHW/TMO5 regulates vascular initial cell production, vascular cell proliferation, and xylem fate determination in the embryo and root apical meristem (Ohashi-Ito et al., 2019). Furthermore, the TMO5/LHW complex is limited to xylem cells and induces production of cytokinin to diffuse into neighboring procambium cells, promoting cell proliferation by induction of DOF-type transcription factors (Miyashima et al., 2019; Smet et al., 2019). The DOF transcription factors, *HCA2*, *TMO6* (*DOF5.3*), *DOF2.1*, and *DOF6*, are rapidly activated at the *Arabidopsis* graft junction. Grafting with the quadruple mutant, *hca2*, *tmo6-4*, *dof2.1-1*, *dof6-1*, reduce phloem reconnection, xylem reconnection, and root growth after grafting. Overexpression of *HCA2*, *TMO6*, *DOF2.1*, or *DOF6* below the graft junction accelerated the rate of phloem reconnection (Zhang et al., 2022). *WOX4* (downstream of the TDIF-PXY module) is responsible for promoting cambium proliferation. Down-regulation of *WOX4* expression by RNA interference in *Arabidopsis* generated small plants that exhibited severe reductions in differentiated xylem and phloem (Ji et al., 2010). Overexpression of *SlWOX4* generates over proliferation of xylem and phloem in transgenic tomato seedlings (Ji et al., 2010). These above genes are mainly involved in the proliferation and differentiation of cambium and xylem. Moreover, our protein interaction analysis reveals that LHW, DOF, ARF4, and other proteins form a protein interaction network to commonly influence the secondary development of rapeseed stems. Additionally, the majority of the genes encoding these were highly expressed in ZS11. Thus, we speculate that the vascular cambium in ZS11 stems is more actively involved in secondary differentiation, resulting in thicker xylem cell layers and denser interfascicular fibrocytes.

### ZS11 possesses a more beneficial cell wall composition

The alteration of cell wall components, such as cellulose and lignin, impacts the mechanical strength of the stem (Xie et al., 2022). The ZS11 stem possessed a substantially higher concentrations of lignin and cellulose compared with 4D122. GO enrichment analysis showed that several vascular-related processes were significantly enriched at flowering stage. In these processes, 26 kinesin-like proteins were up-regulated in ZS11 (**Figure 6D**). Microtubules are essential in cellulose synthesis. Cellulose synthase complex movement on the plasma membrane is driven by cortical microtubules (Paredez et al., 2006). Kinesin-4 is involved in the transport of non-cellulosic materials through the cortical microtubule and is linked with cell elongation and cell wall dynamics (Zhong et al., 2002; Kong et al., 2015). The *fra1* (*AtKINESIN-4A*) mutation results in altered cellulose microfibril deposition (Zhong et al., 2002). Cellulose microfibril involved in cell wall deposition were found to be spooled out of cellulose-synthesizing enzymes movement within the plane of the plasma membrane (Wightman et al., 2009). We discovered that two Kinesin-4 family genes (*BnaC08G0304600ZS*, and *BnaA09G0469500ZS*) were upregulated expression in ZS11. Meanwhile, cytoskeleton organization was enriched at flowering stage, where four *Arabidopsis MAP70-5* homologs were upregulated in ZS11. *MAP70-5* is specifically expressed during xylem differentiation (Pesquet et al., 2010). Downregulation of *AtMAP70-5* results in reduced inflorescence stem length and diameter, and individual cells are inhibited in their capacity for expansion (Korolev et al., 2007). Conversely, overexpression of *MAP70-5* increases the population of xylem cells with spiral secondary walls (Pesquet et al., 2010).

Lignin is an unordered polymer, providing vital mechanical support in vascular plants (Boerjan et al., 2003). The lignin biosynthetic pathway involves sequential hydroxylation and methoxylation of the aromatic ring, coupled with side-chain reduction of the acid to the alcohol (Dixon and Barros, 2019). The methylation process is catalyzed by CCoAOMT and COMT and is the rate-limiting step in lignin synthesis (Dixon and Barros, 2019; Sekula et al., 2020). In this study, expression analysis of genes related to lignin synthesis showed that *CCoAOMT* (*BnaA01G0157400ZS*) and *COMT* (*BnaC03G0147500ZS*) were significantly upregulated in ZS11. Additionally, the catalytic efficiencies of *COMT* and *CCoAOMT* were significantly correlated with their methyl donor of SAM (Dixon and Barros, 2019). Down-regulation of *PvSAMS* in switchgrass reduced SAM and lignin concentrations, implying an important role of *SAMS* in the methionine cycle, lignin biosynthesis (Li et al., 2022). The concentrations of SAM in ZS11 were higher than that of 4D122 (**Figure 5C**). And then we found a significant increase in SAM concentrations and RPR values after exogenous treatment of 4D122 with SAM (**Supplementary Figure S3**), suggesting that high SAM concentration may be one of the reasons for ZS11 highly stem strength. The GO analysis revealed that SAM synthesis-related genes were more highly expressed in ZS11. SAM, as a sulfur-containing metabolite, is closely related to sulfur supply (Nikiforova et al., 2005). In addition, KEGG enrichment analysis revealed that sulfur metabolism was more active in ZS11 stems (**Supplementary Figure S2**). In light of these analytical findings, we hypothesize that the high SAM concentration in ZS11 stems may have encouraged the buildup of lignin, creating stems that are incredibly resistant to lodging.

## Conclusions

Considering the outcomes of the current study, we found that ZS11 exhibited superior stem mechanical properties. The stronger stem of ZS11 simultaneously contributed to higher lignin and cellulose concentrations and greater interfascicular fibrocyte density. Comparative transcriptome analysis showed significant enrichment DEGs for SAM/Met cycle-related processes, microtubule-associated processes, and cytoskeleton organization process in ZS11 stems. 13 stem anatomy-related genes in all possess of complex interactions, which are closely associated with vascular bundle development. Our findings also contribute to a better understanding of how coordinated promotion of cell wall components and vascular structures improve stem strength and promote breeding for lodging resistance of rapeseed (*Brassica napus*).

## Data availability statement

The datasets presented in this study can be found in online repositories. The names of the repository/repositories and accession number(s) can be found in the article/**Supplementary Material**.

## Author contributions

LR and ZT designed the research. ZT, LR, XW, XD, ZT, XZ, and JL performed experiments and analyzed the data. ZT wrote the paper. HW, JT, and LR supervised and complemented the writing. All authors contributed to the article and approved the submitted version.

## Supplementary material

Supplementary Figure 1 Photograph of ZS11 and 4D122 at maturity stage in the field.

Supplementary Figure 2 KEGG enrichment analysis of consistent DEGs in the two stages. **(A)** Top 20 enriched KEGG terms of DEGs that were up-regulated in the two stages. **(B)** Top 20 enriched KEGG terms of DEGs that were down-regulated in the two stages.

Supplementary Figure 3 Different concentrations of exogenous SAM treatment (100, 1000 μM) of 4D122 line. ZS11 was untreated of exogenous SAM. Untreated 4D122 was used as CK. **(A, B)** RPR, and SAM concentrations of different concentration gradients. SAM was sprayed on the leaves at bolting stage. RPR, and SAM concentration were measured on the sixth internode of stem at flowering stage. Error bars represent standard deviation (100 μM: n=5; 1000 μM: n=6; ZS11: n=10). Different letters indicate significant differences at the different treatments (*p* < 0.05 by multiple comparisons).

Supplementary Figure 4 Venn diagram of cell wall-related genes with ZS11 and 4D122 DEGs in the sixth internode of the stem at the flowering or silique stages.
