# Supplementary figures and images for: Integrating biochemical and anatomical characterizations with transcriptome analysis to dissect superior stem strength of ZS11 (Brassica napus)
Source: Front Plant Sci. 2023 May 9;14:1144892. doi: 10.3389/fpls.2023.1144892 (PMC10203542; doi:10.3389/fpls.2023.1144892)

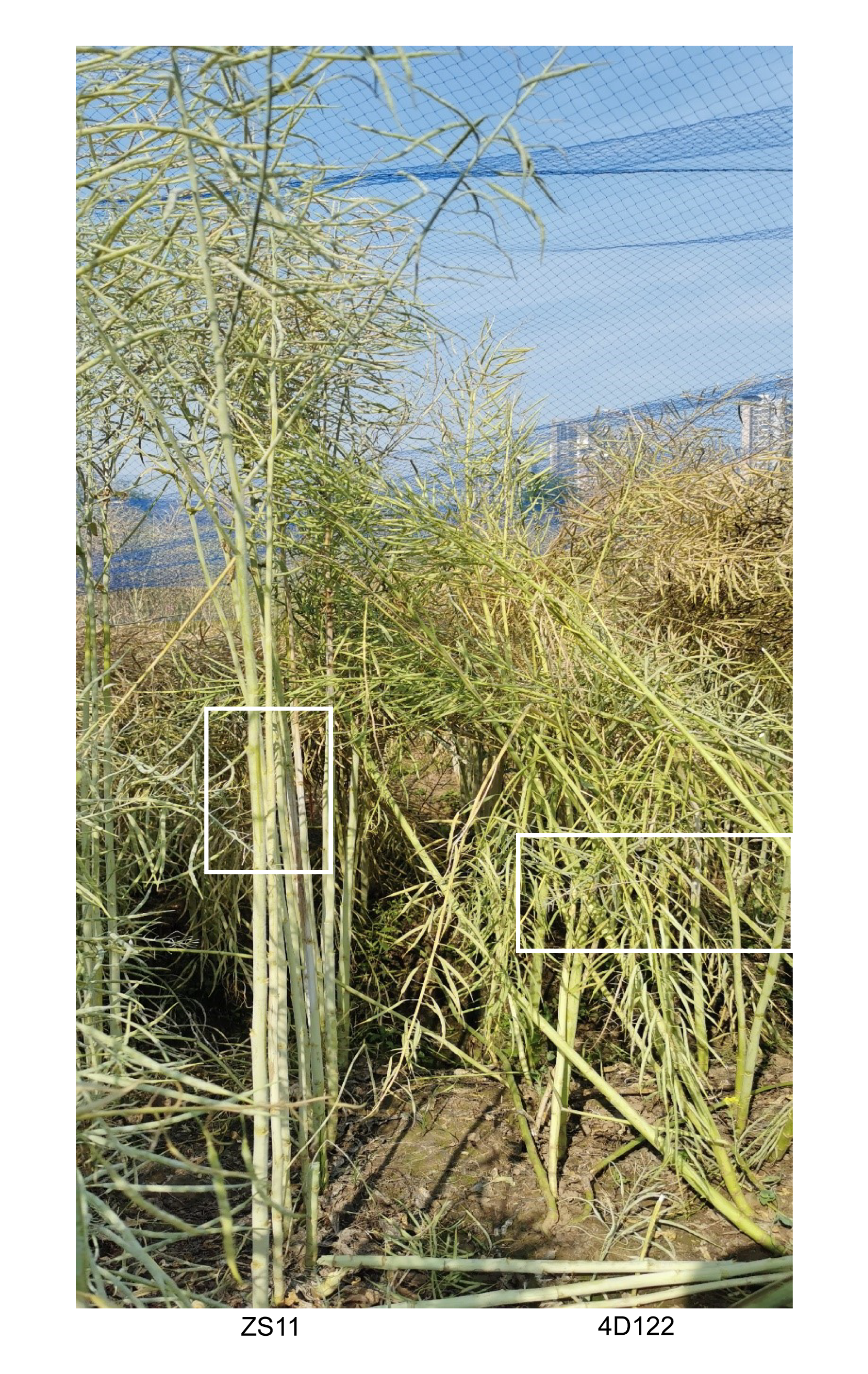

Supplement: Supplementary Figure 1 — Photograph of ZS11 and 4D122 at maturity stage in the field. [file Image_1.tif]

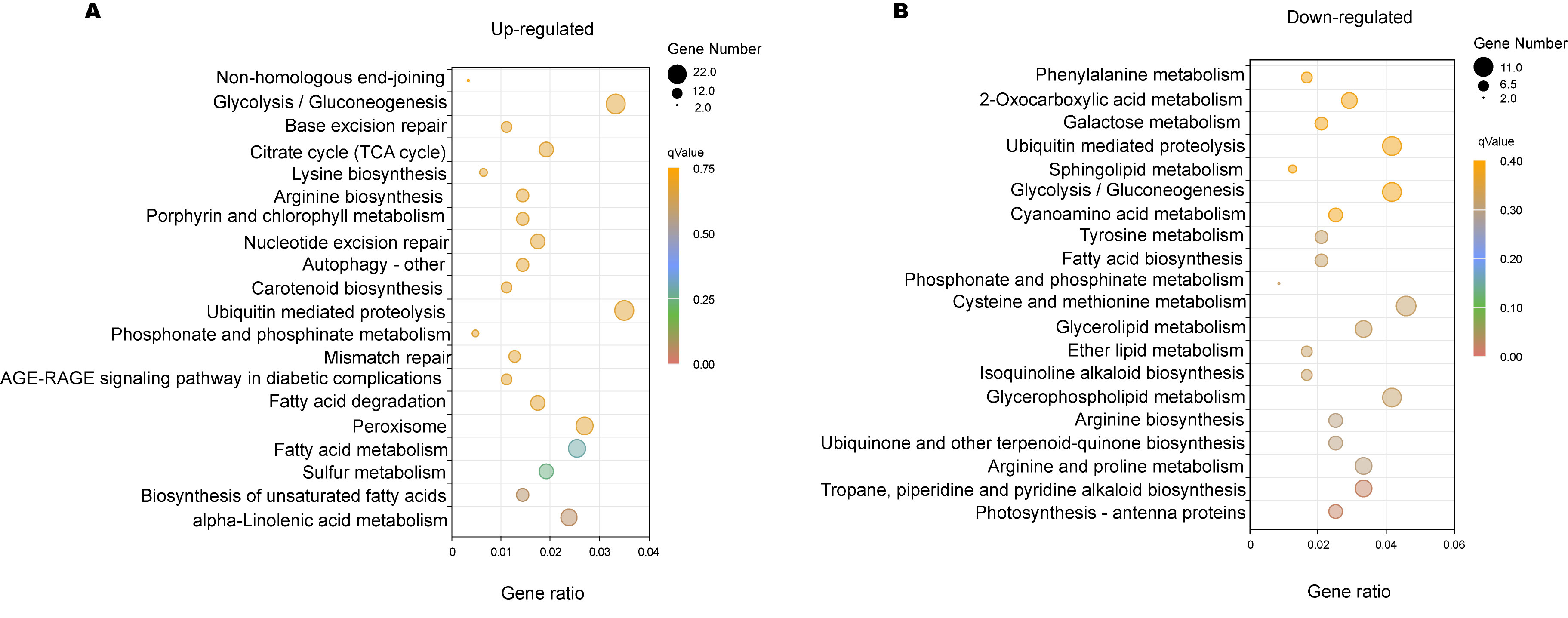

Supplement: Supplementary Figure 2 — KEGG enrichment analysis of consistent DEGs in the two stages. (A) Top 20 enriched KEGG terms of DEGs that were up-regulated in the two stages. (B) Top 20 enriched KEGG terms of DEGs that were down-regulated in the two stages. [file Image_2.jpeg]

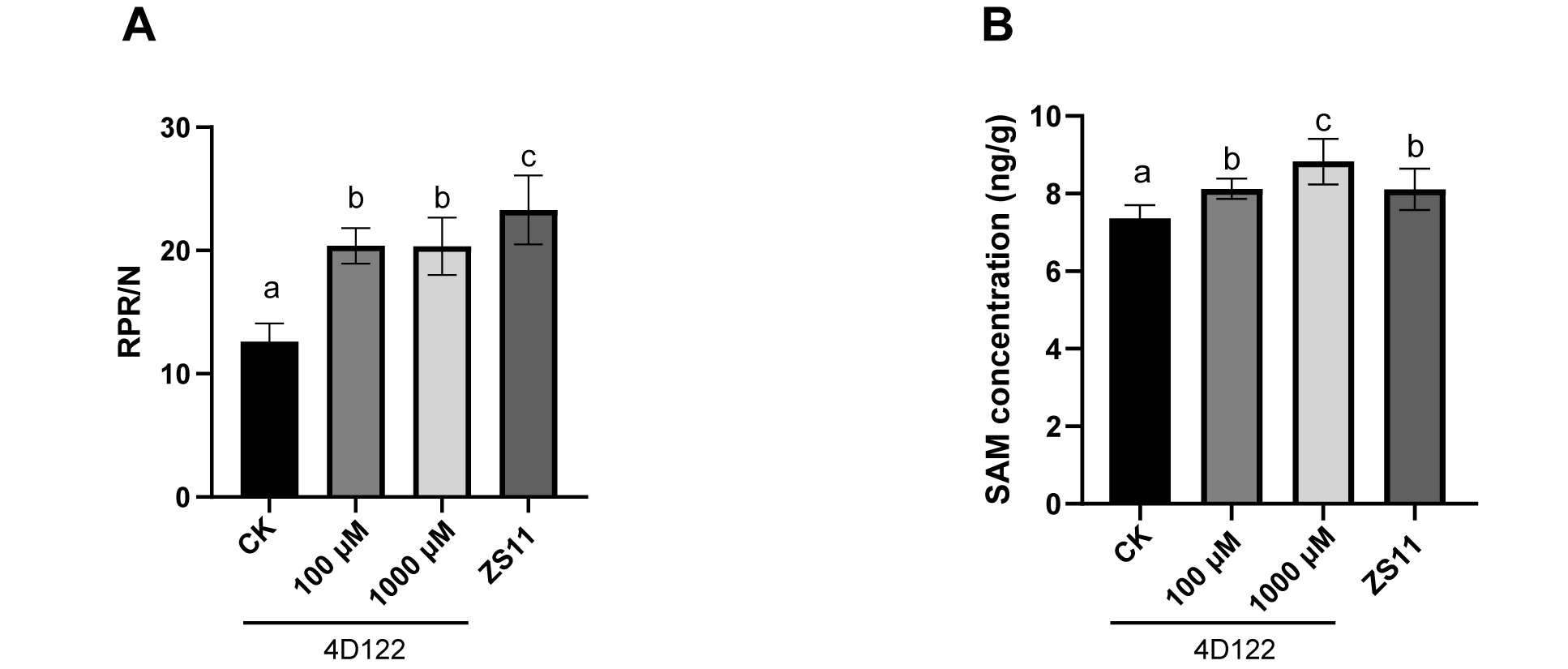

Supplement: Supplementary Figure 3 — Different concentrations of exogenous SAM treatment (100, 1000 μM) of 4D122 line. ZS11 was untreated of exogenous SAM. Untreated 4D122 was used as CK. (A, B) RPR, and SAM concentrations of different concentration gradients. SAM was sprayed on the leaves at bolting stage. RPR, and SAM concentration were measured on the sixth internode of stem at flowering stage. Error bars represent standard deviation (100 μM: n=5; 1000 μM: n=6; ZS11: n=10). Different letters indicate significant differences at the different treatments (p < 0.05 by multiple comparisons). [file Image_3.tif]

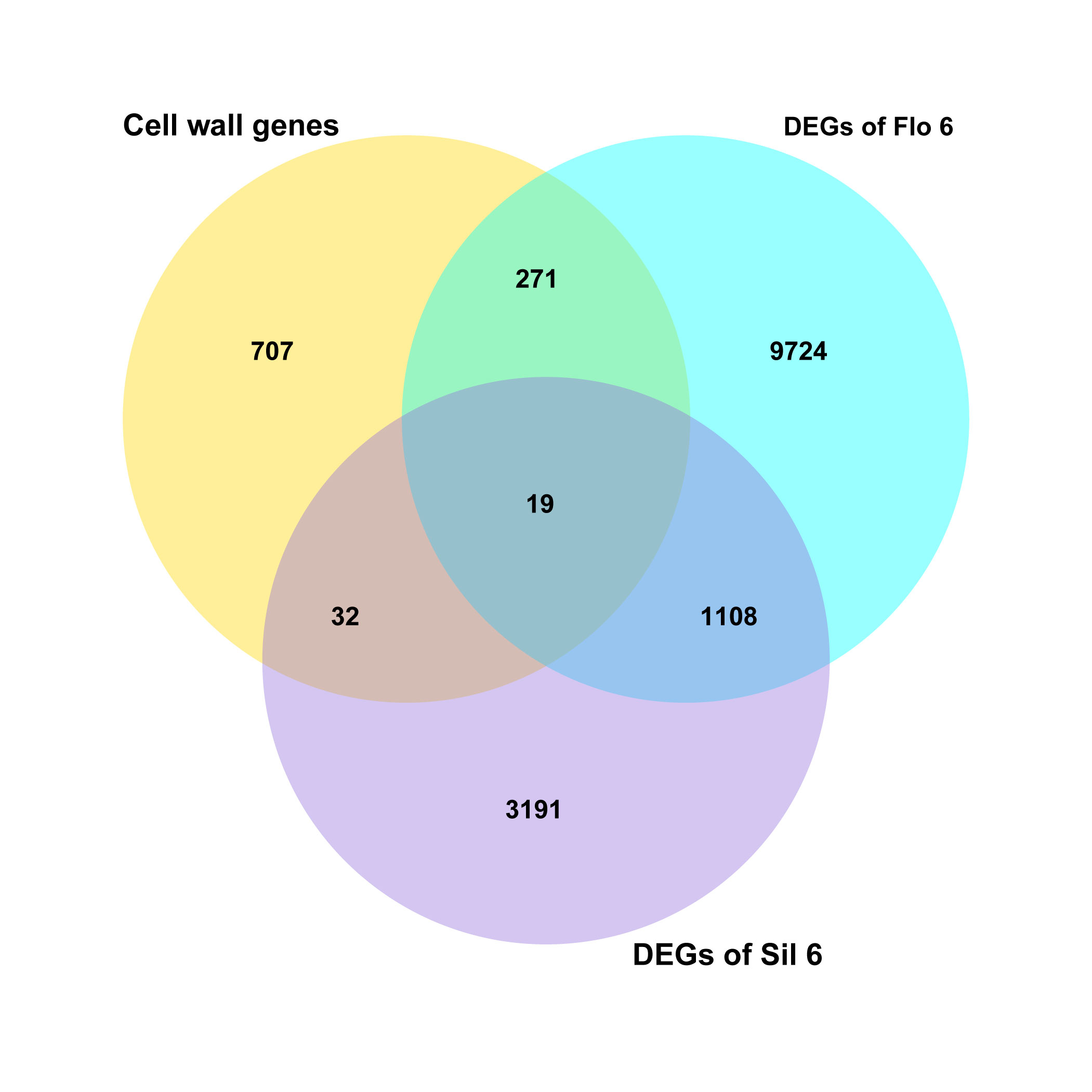

Supplement: Supplementary Figure 4 — Venn diagram of cell wall-related genes with ZS11 and 4D122 DEGs in the sixth internode of the stem at the flowering or silique stages. [file Image_4.jpeg]
